# Direct transformation of *n*-alkane into all-*trans* conjugated polyene via cascade dehydrogenation

**DOI:** 10.1093/nsr/nwab093

**Published:** 2021-05-24

**Authors:** Xuechao Li, Kaifeng Niu, Junjie Zhang, Xiaojuan Yu, Haiming Zhang, Yuemin Wang, Qing Guo, Pengdong Wang, Fangsen Li, Zhengming Hao, Chaojie Xu, Yanning Tang, Zhichao Xu, Shuai Lu, Peng Liu, Guigu Xue, Yen Wei, Lifeng Chi

**Affiliations:** Jiangsu Key Laboratory for Carbon Based Functional Materials & Devices, Institute of Functional Nano & Soft Materials, Soochow University, Suzhou 215123, China; Jiangsu Key Laboratory for Carbon Based Functional Materials & Devices, Institute of Functional Nano & Soft Materials, Soochow University, Suzhou 215123, China; Department of Physics, Chemistry and Biology, IFM, Linköping University, 58183 Linköping, Sweden; Jiangsu Key Laboratory for Carbon Based Functional Materials & Devices, Institute of Functional Nano & Soft Materials, Soochow University, Suzhou 215123, China; Institute of Functional Interfaces, Karlsruhe Institute of Technology, Eggenstein-Leopoldshafen 76344, Germany; Jiangsu Key Laboratory for Carbon Based Functional Materials & Devices, Institute of Functional Nano & Soft Materials, Soochow University, Suzhou 215123, China; Institute of Functional Interfaces, Karlsruhe Institute of Technology, Eggenstein-Leopoldshafen 76344, Germany; Department of Chemistry, Southern University of Science and Technology, Shenzhen 518055, China; Vacuum Interconnected Nanotech Workstation (Nano-X), Suzhou Institute of Nano-Tech and Nano-Bionics, Chinese Academy of Sciences, Suzhou 215123, China; Vacuum Interconnected Nanotech Workstation (Nano-X), Suzhou Institute of Nano-Tech and Nano-Bionics, Chinese Academy of Sciences, Suzhou 215123, China; Jiangsu Key Laboratory for Carbon Based Functional Materials & Devices, Institute of Functional Nano & Soft Materials, Soochow University, Suzhou 215123, China; Jiangsu Key Laboratory for Carbon Based Functional Materials & Devices, Institute of Functional Nano & Soft Materials, Soochow University, Suzhou 215123, China; Jiangsu Key Laboratory for Carbon Based Functional Materials & Devices, Institute of Functional Nano & Soft Materials, Soochow University, Suzhou 215123, China; Jiangsu Key Laboratory for Carbon Based Functional Materials & Devices, Institute of Functional Nano & Soft Materials, Soochow University, Suzhou 215123, China; Jiangsu Key Laboratory for Carbon Based Functional Materials & Devices, Institute of Functional Nano & Soft Materials, Soochow University, Suzhou 215123, China; Vacuum Interconnected Nanotech Workstation (Nano-X), Suzhou Institute of Nano-Tech and Nano-Bionics, Chinese Academy of Sciences, Suzhou 215123, China; Department of Chemistry, Southern University of Science and Technology, Shenzhen 518055, China; Department of Chemistry, Southern University of Science and Technology, Shenzhen 518055, China; Key Laboratory of Organic Optoelectronics and Molecular Engineering, Department of Chemistry, Tsinghua University, Beijing 100084, China; Jiangsu Key Laboratory for Carbon Based Functional Materials & Devices, Institute of Functional Nano & Soft Materials, Soochow University, Suzhou 215123, China

**Keywords:** C(sp3)–H activation, alkane transformation, on-surface synthesis, dehydrogenation, molecular wiring

## Abstract

Selective C(sp^3^) −H activation is of fundamental importance in processing alkane feedstocks to produce high-value-added chemical products. By virtue of an on-surface synthesis strategy, we report selective cascade dehydrogenation of *n*-alkane molecules under surface constraints, which yields monodispersed all-*trans* conjugated polyenes with unprecedented length controllability. We are also able to demonstrate the generality of this concept for alkyl-substituted molecules with programmable lengths and diverse functionalities, and more importantly its promising potential in molecular wiring.

## Teaser

Direct transformation of n-alkanes and alkyl-substituted derivatives has been achieved under surface constraints to yield conjugated polyenes with programmed length and functionalities.

## INTRODUCTION

The utilization of alkane feedstocks in synthesis is mainly restricted by the chemically inert C(sp^3^) −H bonds, which appeals for preliminary functionalization to enhance their chemical selectivity [1–5]. Advances of direct alkane dehydrogenation on heterogeneous catalysts have been demonstrated in petrochemistry to produce high-value-added mono-olefins or dienes where light alkanes are usually the major concern [6,7] in this energy-consuming reaction. As the number of carbon atoms increases, dehydrogenation of heavy alkanes (known as paraffins) causes loss of chemical selectivity to construct more C=C bonds without suffering uncontrolled side reactions such as isomerization, cyclization and aromatization [8]. The complicated reaction pathways in consecutive paraffin dehydrogenation impede comprehension of the related alkane activation, concealing any inherent order involved in the production of double bonds and making complete transformation of paraffins to polyenes an unachievable objective.

The rise of on-surface chemistry over the last decade provides unprecedented strategies to address selective C–H bond activation, allowing for precise fabrication of novel carbon-based functional nanostructures [9–11]. Combined with on-site, versatile microscopic and spectroscopic characterizations, intensively explored on-surface synthesis has also demonstrated hidden chemical details in the heterogeneous catalytic process far beyond the structures [12–16].

**Figure:**
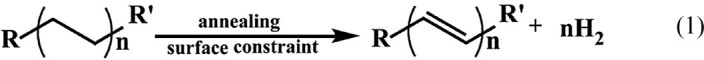


Herein, we demonstrate a highly selective on-surface transformation which directly transforms *n*-alkane molecules into the corresponding all-*trans* conjugated polyenes (Formula (1)). The formation of conjugated C=C bonds was examined with versatile characterization skills including scanning tunneling microscopy and non-contact atomic force microscopy (STM/nc-AFM), infrared reflection adsorption spectroscopy (IRRAS), temperature programmed desorption (TPD) as well as angle-resolved photoemission spectroscopy (ARPES). Combined with density functional theory (DFT) simulations, we also managed to clarify a cascade mechanism in this novel chemical transformation in which the steric effect derived from strict surface epitaxy plays an essential role. The developed strategy is also applicable for various alkyl-substituted precursors with depressed energy consumption down to room temperature, which endows the derived polyenes with programmable lengths, functionalities as well as the possibility to integrate either homogeneous or heterogeneous functional moieties with conducting polyene wires. We expect that the strategy proposed here will broaden the developed methodology of on-surface synthesis, and have brilliant prospects for construction of ideal molecular topology to spur investigations in the surface science and organic electronic/semiconductor physics community.

## RESULTS AND DISCUSSION

**Figure 1.**
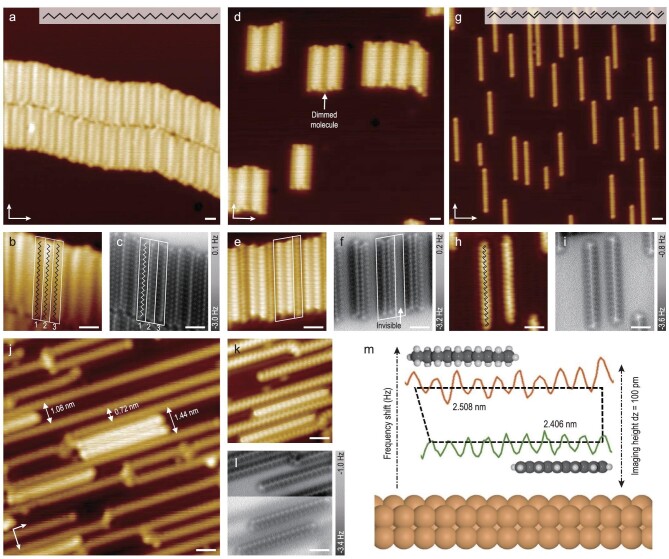
STM/nc-AFM investigation towards polyene transformation on Cu(110). (a) STM image (−200 mV, 3 pA) of self-assembled *n*-C_32_H_66_ molecules on Cu(110). (b and c) Close-up STM image (−200 mV, 5 pA) and corresponding nc-AFM image of (a) at dz = 180 pm with respect to the STM offset. The superstructure unit cell is labeled in a white box superimposed by the skeletal formula of *n*-C_32_H_66_ molecules. (d) STM image (−50 mV, 8 pA) of the sample after thermal annealing under 453 K for an hour. (e and f) Close-up STM image (−5 mV, 8 pA) of (d) and the corresponding nc-AFM image at dz = 130 pm with respect to the STM offset with the new superstructure labeled in a white box. (g) Low-flux deposition of *n*-C_32_H_66_ on Cu(110) held at 453 K for 30 minutes. (h and i) Close-up STM image (−5 mV, 8 pA) of [32]-polyene superimposed with the skeletal formula and the corresponding nc-AFM image at dz = 30 pm with respect to the STM offset. (j) STM image (−1 V, 100 pA) of polymerized polyenes after annealing a high coverage *n*-C_32_H_66_ sample under 493 K for an hour with the regular intervals labeled. (k and l) Close-up STM image (−5 mV, 8 pA) of (j) and the corresponding nc-AFM image for transformed polyenes and intact *n*-C_32_H_66_ molecules under different imaging heights. (m) Periodicity measurements illustrated by a graphical model of *n*-decane and [10]-polyene. The intercepted 10-units cross-section profile from *n*-C_32_H_66_ (orange) and [32]-polyene (green) are displayed for comparison. The white scale bars are 1 nm, referring to all the STM/nc-AFM images.

Molecular deposition of *n*-dotriacontane (*n*-C_32_H_66_) on Cu(110) held at room temperature results in a self-assembled lamellar structure with molecular long-axis aligning along the [$1\bar{1}0$] direction of the substrate (Fig. 1a). Lattice incommensuration between the molecules and the substrate introduces a superstructure containing three *n*-C_32_H_66_ molecules (labeled as molecules I, II and III in Fig. 1b) in a unit cell whose periodicity is measured to be 14.7 Å (corresponding to nearly four rows of Cu(110) ridges). A combined STM/nc-AFM investigation indicates that the *n*-C_32_H_66_ molecules adsorb on Cu(110) with the zigzag carbon skeleton almost parallel to the surface (Fig. 1c). The outermost hydrogen atoms of methylene groups contribute a brighter ball-shaped appearance in nc-AFM imaging [17]. Although these molecules lie almost flat on the surface, the non-uniform frequency shift distribution above molecules II and III within the superstructure indicates a slightly tilted adsorption geometry compared with molecule I. In reference to the underpinning copper atoms, only the flat-on *n*-C_32_H_66_ molecule within the superstructure (molecule I) settles right on the groove of Cu(110), while the other two tilt-on molecules mismatch the ridges to varying degrees (Supplementary Fig. S1). Upon annealing the sample at 453 K for an hour, the self-assembled lamellae are observed to disperse on the surface (Fig. 1d), presenting a similar superstructure (14.4 Å) where one of every three paraffin chains differentiates from the others in STM contrast. The dimmed species observed in the close-up STM image (Fig. 1e), however, are not distinguishable in nc-AFM characterization at the typical imaging height of *n*-C_32_H_66_ molecules (Fig. 1f). Aside from the dimmed molecule, the remaining two molecules within the new superstructure remain intact where the ball-shaped alkane feature in the frequency shift channel preserves along the chains.

To fully recognize these ‘disappeared’ species, a low-flux deposition (30 minutes) experiment of *n*-C_32_H_66_ is conducted on a hot Cu(110) substrate held at 453 K. Figure 1g displays well-isolated molecules with preferential adsorption on the Cu(110) ridges (Supplementary Fig. S2) presenting almost homogeneous length distribution. The typical ball-shaped feature of alkanes in the frequency shift channel is now replaced by the remarkable line contrast (Fig. 1h and i) as a result of the in-plane stretched C–H bonds of conjugated alkenes [18]. The difference in molecular appearance therefore implies a possible, but unexpected, thorough chemical transformation from saturated alkanes into the corresponding all-*trans* conjugated polyenes where hydrogen atoms are selectively scissored. The derived molecules from *n*-C_32_H_66_ are named (3*E*,5*E*,7*E*,9*E*,11*E*,13*E*,15*E*,17*E*,19*E*,21*E*,23*E*,25*E*,

27*E*,29*E*)-dotriaconta-1,3,5,7,9,11,13,15,17,19,21,

23,25,27,29,31-hexadecaene according to IUPAC nomenclature. But for convenient discussion, we term the transformed molecules as [n]-polyene where ‘n’ is the number of carbon atoms of the conjugated polyene chain referred to the nomenclature of annulenes [19].

After annealing a full monolayer *n*-C_32_H_66_ sample at 493 K for an hour, polymerization among [32]-polyene species prevails and results in long polyene chains over the terrace (see Supplementary Fig. S3 for more details). The transformed polyenes are separated by regular intervals (Fig. 1J) that are nearly the integral multiple of Cu(110) row periodicity (0.36 nm) because of their ridge-preferred adsorption behavior. In spite of the chemical reactivity of all C=C bonds, intermolecular couplings are constrained at the terminal alkenes [20] on the same ridge or neighboring ridges, which might be because of their surface-protected adsorption [21] and confined migration [22] on Cu(110). All-*trans* interconnectivity dominates as illustrated by the homogeneity in STM imaging, while the defects (bright nodes in STM) within the polymerized chains are recognized by nc-AFM to be *cis*-isomerization (Supplementary Fig. S4).

**Figure 2.**
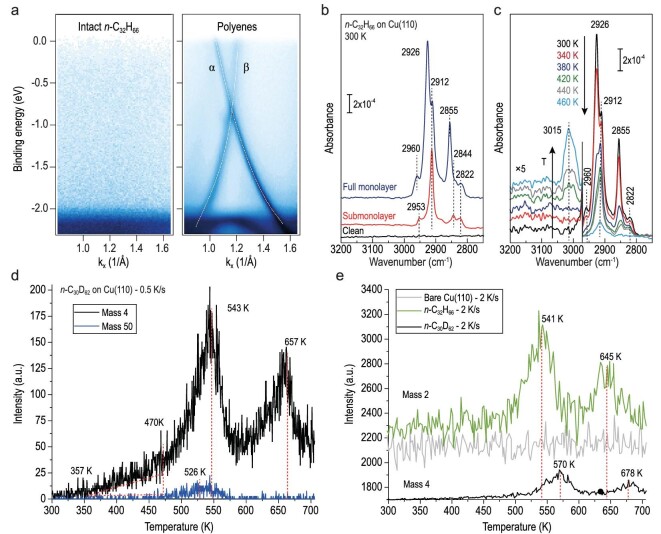
Complementary evidence of the chemical transformation from *n*-alkane into [n]-polyene. (a) Band dispersion near Y point of intact paraffin molecules (left) and polymerized polyenes (right) under room temperature. The two polyene-related bands α and β were highlighted by white dashed lines. (b) Coverage-dependent IRRAS data of *n*-C_32_H_66_ molecules deposited on Cu(110) at 300 K. (c) Temperature-resolved IRRAS data of *n*-C_32_H_66_ molecules after full monolayer adsorption on Cu(110) at 300 K and then annealing gradually to higher temperatures (up to 460 K and stay for an hour). (d) TPD signals of the deuterium gas (D_2_, *m/z* = 4) and propyl fragments (*m/z* = 50) collected from a fully covered *n*-C_30_D_62_ sample under a heating rate of 0.5 K/s. (e) Comparison between TPD signals of deuterium gas (black, D_2_, *m/z* = 4) and hydrogen gas (green, H_2_, *m/z* = 2) under a heating rate of 2 K/s in reference to the bare Cu(110) baseline (gray).

The developed π-conjugation remarkably enhances the molecule–substrate interaction, thus decreasing the adsorption height as a result. Experimentally, an extra tip approaching process by 100 pm compared with the typical imaging height of paraffin molecules is found necessary to clearly visualize the carbon skeleton of transformed polyenes, as depicted in Fig. 1k and l. The periodicity along the long axis of the transformed species in nc-AFM measurement is 2.41 ± 0.02 Å (the green line in Fig. 1m), while the periodicity along the *n*-C_32_H_66_ molecules is determined to be 2.51 ± 0.02 Å (the orange line in Fig. 1m). These values correspond well with the early X-ray diffraction data [23,24] and our DFT calculations where the simulated periodicity for [10]-polyene and *n*-decane are 2.47 Å and 2.56 Å, respectively. The periodicity measured here is also consistent with the recently reported nc-AFM investigation on the individual polyacetylene chain derived from direct acetylene polymerization on Cu(110) [25]. Similar metallic band dispersion apparently evolves (Fig. 2a) after the insulating *n*-C_32_H_66_ layer is transformed into polymerized polyene scaffolds in our angle resolved photoemission (ARPES) measurements (see more in Supplementary Fig. S5).

**Figure 3. fig3:**
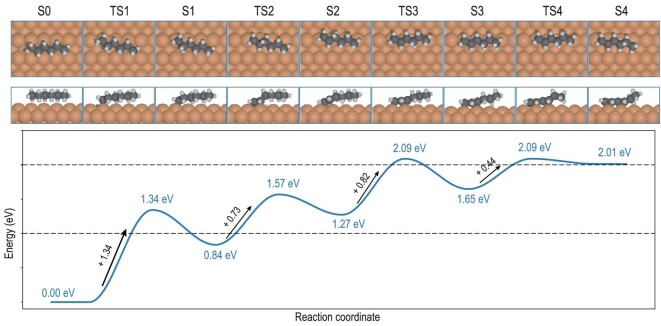
Cascade dehydrogenation of *n*-alkanes on Cu(110) illustrated by DFT simulations. The overall pathway and energy profile for consecutive dehydrogenation process of *n*-hexane. The Cu, C and H atoms are represented by the orange, gray and white circles, respectively.

Temperature-resolved infrared reflection absorption spectroscopy (IRRAS) was used to provide chemical evidence for Cu-catalyzed thermal conversion of saturated methylene (C(sp^3^)H_2_) groups to unsaturated C(sp^2^)H groups. As illustrated by the red curve in Fig. 2b, the adsorption of sub-monolayer *n*-C_32_H_66_ on Cu(110) at 300 K is characterized by an intense vibration at 2912 cm^–1^ and a low-lying band at 2822 cm^–1^. The latter is a fingerprint for the softened ν(C−H_proximal_) vibration perturbed by the interaction between H atoms and surface Cu atoms, while the 2912 cm^–1^ band is ascribed to the ν(C−H_distal_) mode of dangling C–H groups of adsorbed *n*-C_32_H_66_ in a flat-on configuration [26]. After saturated deposition (full ML) of *n*-C_32_H_66_ at 300 K (blue curve in Fig. 2b), both the asymmetric (ν_as_(CH_2_), 2926 cm^–1^) and symmetric (ν_s_(CH_2_), 2855 cm^–1^) vibrations show up and shift to higher frequency as a consequence of the condensed molecular packing. When subjecting the *n*-C_32_H_66_-saturated Cu(110) surface to annealing at elevated temperatures, we observe first the degradation of IR bands at 2926 and 2855 cm^–1^. Further annealing the sample to 420 K leads to attenuation of the IR signals at 2912 and 2822 cm^–1^ (flat-on *n*-C_32_H_66_), but a weak band at 3015 cm^–1^ appears simultaneously which is characteristic for the stretching vibration of C(sp^2^)–H bonds for all-*trans* polyenes [27]. The intensity of this newborn signal proceeds with increasing temperatures and becomes saturated over 460 K while the methylene-related vibrations almost completely vanish (Fig. 2c),3 revealing the direct conversion of paraffin into conjugated polyene via selective dehydrogenation. We propose that the rather weak signal around 3015 cm^–1^ is attributed to the flat-on geometry of the polymerized polyene molecules, in which the transition dipole moment of the C–H vibrations is oriented virtually parallel to the surface (i.e. almost IR-inactive based on the surface selection rule [28]).

The selective dehydrogenation was further monitored by complementary temperature programmed desorption (TPD) experiments in which deuterated triacontane (*n*-C_30_D_62_) is selected to avoid the H_2_ contribution from the background. After depositing a full monolayer of *n*-C_30_D_62_ molecules, TPD signals of deuterium gas (D_2_, *m/z* = 4) and *n*-C_30_D_62_ (propyl fragments, mass = 50) were collected (Fig. 2d). Two desorption peaks are observed in the TPD trace of *m/z* = 4. The first desorption peak starts at about 357 K, and gives a peak at 543 K. Meanwhile, the apparent desorption of *n*-C_30_D_62_ appears near 480 K and peaks at 526 K. The difference in desorption signal evolution (300–600 K) between D_2_ and propyl fragments implies the prominent dehydrogenation process in polyene formation as discussed above. The second peak of D_2_ at 657 K is assumed to be the consequence of polyene reforming where alkene dehydrogenation dominates. The effect of deuterium substitution is examined by comparing the TPD results of *n*-C_32_H_66_. Considering the high background of H_2_ in the UHV chamber, the baseline of the TPD trace of D_2_ was increased for comparison (black curve in Fig. 2e). It is obvious that the reaction-related desorption of H_2_ (or D_2_) shifts to a higher temperature for deuterium substitution. Moreover, raising the heating rate from 0.5 K/s to 2 K/s would postpone both the dehydrogenation peaks by about 20 K for *n*-C_30_D_62_ while enhancing the signal-to-noise ratio in return.

**Figure 4. fig4:**
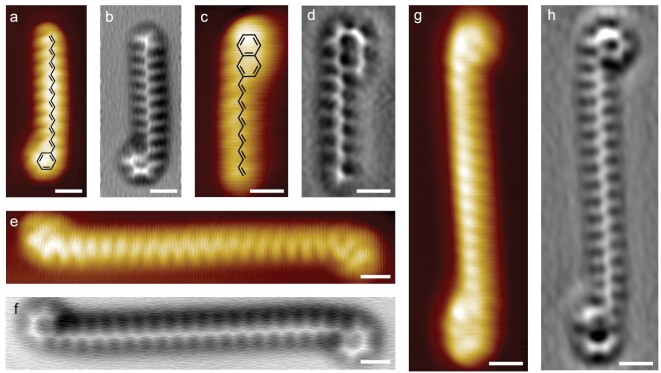
Generality of polyene transformation and the possibility for molecular wiring. (a–d) Chemical formula, STM image (−5 mV, 8 pA) and Laplacian-filtered AFM image of transformed products of octadecylbenzene and 2-dodecylnaphthalene. (e–h) STM images (−5 mV, 8 pA) and the corresponding Laplacian-filtered AFM images of homogeneously (e and f) and heterogeneously (g and h) coupled polyene motifs with the products in (a–d). The white scale bars are 0.5 nm referring to all the STM/nc-AFM images.

The consecutive dehydrogenation of paraffin molecules was often thought to be a chaotic reaction involving miscellaneous competing pathways. To completely understand this selective C–H bond scission in *n*-C_32_H_66_ transformation on Cu(110), DFT calculations were applied to scrutinize the dehydrogenation process step by step as demonstrated in Fig. 3. For simplification,4 we start with a single *n*-hexane molecule (S0) physiosorbed on the grooves of Cu(110) along the [$1\bar{1}0$] direction [29]. The Cu atoms on surface serve as the active sites suggesting a ‘surface-stabilized’ reaction pathway [30]. For *n*-hexane, the first C–H activation may have three alternative starting points, namely C1, C2 and C3 sites. The activation energy of the terminal C–H bond is calculated to be 1.34 eV (S0 to S1), while the energy barriers for dehydrogenation at C2 and C3 sites are both above 1.50 eV (Supplementary Fig. S6). C–H activation at the C1 (−CH_3_) site is therefore the most energetically favorable pathway as reported previously [31]. Passing through TS1, the 1st dehydrogenation finalizes with the 1-hexyl termination connecting the ridge Cu atoms and leaves the dissociated H atom on the surface (S1). The subsequent dehydrogenation barrier at the C2 site is calculated to be 0.73 eV (TS2), which induces the terminal olefin formation (S2). Thereafter, the hydrogen cleavage at both C3 and C4 sites (TS3 and TS4) requires comparatively low activation energies (0.82 and 0.44 eV, respectively) for double bond formation, which further throws the skeleton towards the surface ridges. As the initiation barrier at the C1 site is higher, the (*E*)-1,3,5-hexatriene ([6]-polyene) would be finally generated epitaxially above the cooper ridges via consecutive dehydrogenation in a cascade process [32]. The yield of polyene transformation in a single chain manner may still be limited by the C–C coupling after C–H activations through which alkane polymerization competes [11]. Our DFT calculations, however, indicate that once the terminal C–H in *n*-hexane is activated, the dehydrogenated radicals would prefer to undergo consecutive dehydrogenations on Cu(110) surface rather than coupling with the other one, which is contradictory to the case on Au(110) (see more details in Supplementary Fig. S6). The discrepancy in optimized adsorption before and after the transformation also raises the question of whether the dehydrogenation of alkane occurs initially at the optimized groove sites or directly above the catalytic copper rows because of steric molecular self-assembly. According to the adsorption configuration of *n*-C_32_H_66_ determined experimentally, we construct an optimized supercell of three *n*-hexane molecules assigned to three different adsorption sites: (I) ridge, (II) ridge/groove and (III) groove. Compared with the groove sites, the ridge sites present even enhanced catalytic performance ascribed to the declined distance between the carbon atoms and the catalytic cooper row atoms (Supplementary Fig. S6). This is also consistent with the STM results of the full monolayer sample as presented in Supplementary Fig. S3, where molecular motion is sterically hindered.

The transformation occurring in *n*-alkanes is also available for alkyl-substituted molecules, for which a much lower activation barrier is observed. The complete transformation of octadecylbenzene (OB) experiences a temperature drop by 60 K compared with *n*-C_32_H_66_ (Fig. 4a and b and Supplementary Fig. S7). As the aromatic system extends from benzene to naphthalene, the polyene transformation even proceeds under several hours at room temperature annealing for 2-dodecylnaphthalene (2-DN) (Fig. 4c and d and Supplementary Fig. S8). The depressed energy consumption for alkyl-substituted molecules is attributed to the anchoring effect of substituents that pull the alkyl chains downwards to the surface and initiate alkane dehydrogenation from the substituent site rather than the terminal methylene groups (Supplementary Fig. S9). Therefore, it is also feasible that polyene transformation occurs on other copper surfaces (e.g. Cu(111) as illustrated in Supplementary Fig. S10) through rational precursor selection.

Despite the dramatic decline in activation barrier of alkyl aromatics, the temperature threshold for alkenyl homocoupling on Cu(110) of different polyene species shares a fairly similar starting point at 453 K, far below the reported onset of benzene dehydrogenation [33]. Through the selective alkenyl homocoupling discussed above, it would therefore be convenient to construct complicated polyene motifs that are hardly accessible in solution synthesis, using either homogeneous or heterogeneous precursors with programmable lengths of conjugation as well as diverse functionalities at terminals (Fig. 4e–h). Besides preparing miraculous molecules, it is also a promising strategy to integrate functional molecules systematically into molecular circuits as polyenes can serve as an effective electron transfer medium. Although benzene and naphthalene are unobtrusive functional substituents, they are still sufficient to demonstrate the prototype of diverse molecule integration based on this paraffin-polyene transformation strategy.

## CONCLUSION

With complementary microscopic and spectroscopic characterizations, we clarified a cascade process of alkane dehydrogenation under surface constraints that directly transform *n*-alkane molecules into the corresponding all-*trans* conjugated polyenes. The proposed strategy is further applicable for diverse alkyl substituted molecules with depressed energy consumption above different crystallographic planes of copper surfaces. Combined with hierarchical on-surface reactions, it is feasible to integrate molecular functional units and construct extremely complicated molecules in conjugation to spur further investigations in molecular circuits, molecular topology as well as organic superconductivity [34]. We also anticipate that our investigations in the context together with the clarified steric effect in the transformation could provide a novel perspective on use of alkane feedstocks and become constructive in design of real catalysts by applying for example nanocrystalline and zeolites, which may even trigger up-scaled implications in petrochemical processing.

## Supplementary Material

nwab093_Supplemental_File
