# Supplementary material for: Direct transformation of n-alkane into all-trans conjugated polyene via cascade dehydrogenation
Source: Natl Sci Rev. 2021 May 24;8(10):nwab093. doi: 10.1093/nsr/nwab093 (PMC8566175; doi:10.1093/nsr/nwab093)

Supplementary Materials for

**Direct transformation of *n*-alkane into all-*trans* conjugated polyene via cascade dehydrogenation**

Xuechao Li^1^*, Kaifeng Niu^1, 6^*, Junjie Zhang^1^*, Xiaojuan Yu^2^*, Haiming Zhang^1†^，Yuemin Wang^2†^, Qing Guo^3^, Pengdong Wang^4^, Fangsen Li^4^, Zhengming Hao^1^, Chaojie Xu^1^, Yanning Tang^1^, Zhichao Xu^1^, Shuai Lu^1, 4^, Peng Liu^3^, Guigu Xue^3^, Yan Wei^5^, Lifeng Chi^1†^

^1^ Jiangsu Key Laboratory for Carbon Based Functional Materials & Devices, Institute of Functional Nano & Soft Materials (FUNSOM), Soochow University, Suzhou 215123, Jiangsu, People’s Republic of China

^2^ Institute of Functional Interfaces (IFG), Karlsruhe Institute of Technology (KIT), Eggenstein-Leopoldshafen 76344, Germany

^3^ Department of Chemistry, Southern University of Science and Technology, Shenzhen, Guangdong 518055, People’s Republic of China

^4^ Vacuum Interconnected Nanotech Workstation (Nano-X), Suzhou Institute of Nano-Tech and Nano-Bionics (SINANO), Chinese Academy of Sciences (CAS), Suzhou 215123, Jiangsu, People’s Republic of China

^5^ Key Laboratory of Organic Optoelectronics and Molecular Engineering, Department of Chemistry, Tsinghua University, Beijing 100084, People’s Republic of China

^6^ Department of Physics, Chemistry and Biology, IFM, Linköping University, 58183 Linköping, Sweden

*These authors contributed equally to this work.

†Corresponding author. Email: [chilf@suda.edu.cn](mailto:chilf@suda.edu.cn), [hmzhang@suda.edu.cn](mailto:hmzhang@suda.edu.cn), [yuemin.wang@kit.edu](mailto:yuemin.wang@kit.edu)

**Methods**

**STM/NC-AFM Measurements:** The experiments were conducted in a commercial LT-STM (Scienta Omicron, Germany) operated at 4.6 K under a base pressure of 2.0 × 10^-11^ mbar. A grounded qPlus tuning fork attached with a tungsten tip (a resonance frequency f_0_ ≈ 25.6 kHz, a quality factor Q > 10000 and oscillation amplitude A = 60 pm) was operated in the frequency-modulated mode. The AFM images were recorded in the constant-height mode at zero bias with a CO functionalized termination. The CO tip used for bond imaging on Cu(110) was initially prepared on Cu(111) by exploiting Bartel’s method. [1] The tip-height offsets (dz) for constant-height AFM images were defined relative to the STM setpoint where the positive (or negative) values indicate an increasing (or decreasing) tip-sample distance with respect to the STM offset. The single crystalline substrates (Mateck, Germany) were prepared through cycles of sputtering and annealing up to 800 K. The molecules were evaporated onto the substrate held at room temperature using a commercial Knudsen cell from Kentax (Germany).

**DFT simulations:** All density functional theory (DFT) calculations were performed by using Vienna Ab-initio Simulation Package (VASP) together with the Atomic Simulation Environment (ASE). [2,3] The projector augmented wave (PAW) potentials were employed to describe the electron-ion interactions. [4] The exchange-correlation interactions were treated by Perdew-Burke-Ernzerhof functions (PBE) of generalized gradient approximation (GGA) pseudopotentials. [5] The van der Waals interactions were described by vdW-D3 method developed by Grimme. [6] The cutoff energy for the plane wave basis was set as 400 eV. The periodic interactions were avoided by employing a vacuum layer of 20 Å. The structures of all local minima were optimized until the residual forces on atoms were below 0.02 eV/Å. The dehydrogenations of the *n*-dotriacontane (*n*-C_32_H_66_) were modeled by the successive C−H activations of the *n*-alkane (C_6_H_14_) on the groove of Cu(110) surface, in which the Cu(110) surface was modeled by the periodic slab consisting of four layers. The transition states search for C−H activations was first calculated by the Climb-Image Nudged Elastic Band (CI-NEB), where 10 images were inserted between the initial and final states. [7,8] Subsequently, the central images were used as the input of the Dimer method to obtain transition states. [9] The Brillouin zone of the reciprocal lattice was modeled by gamma-centered Monkhorst-Pack scheme, in which the Γ point and 2 × 2 × 1 grid were adopted for all calculations. [10] In the process of cascade dehydrogenations, the dissociated H atoms bind to the Cu surface. The energy along the reaction path is defined as:

$$\Delta E= E_{Sn}-E_{S0}+nE_{ad-H},$$

where the $E_{Sn}$is the total energy of corresponding transition, intermediate, or final state, and $E_{S0}$ is the total energy of the initial state. The integer *n* represents the number of H atoms dissociated from the molecule. The $E_{ad-H}$ is the reference energy for a single H atom adsorbed on the Cu(110) surface.[11]

**IRRAS measurements:** The IRRAS experiments were performed in an advanced UHV apparatus (Prevac), which combines a state-of-the-art FTIR spectrometer (Bruker Vertex 80v) with several other surface-sensitive techniques (XPS and LEED) as described elsewhere. [12] The Cu(110) single crystal was cleaned by cycles of Ar^+^-sputtering and annealing up to 800 K. Additionally, the cleanliness of the sample was monitored by XPS. The molecules were evaporated onto the substrate held at room temperature using a commercial Knudsen cell from Kentax. The IRRAS data were accumulated at a fixed grazing incidence angle of 80° by recording typically 1024 scans with a resolution of 4 cm^-1^. Prior to each exposure, a spectrum of a clean sample was recorded as a background reference. The base pressure during acquisition of IRRAS data was below 1 × 10^-10^ mbar.

**ARPES measurements:** Angle resolved photoemission spectroscopy (ARPES) experiments were performed at NANO-X lab in Suzhou Institute of Nano-Tech and Nano-Bionics, CAS, with VG DA30L analyzer and He VUV light source of which the energy and angular resolutions are better than 30 meV and 0.1 degree, respectively. The samples were prepared and inspected with the LT-STM facility and then transferred for ARPES measurements with UHV suitcase (Cryoscan, France) which is pumped by a non-evaporable getter (NEG) pump to maintain a base pressure of 1 × 10^-10^ mbar.

**TPD measurements:** The temperature programmed desorption (TPD) experiments were carried out on a home-built TPD-photocatalysis apparatus at Southern University of Science and Technology (SUSTech) as described in Ref. [13]. The Cu(110) single crystal (Mateck, Germany) was cleaned by cycles of Ar^+^-sputtering and UHV annealing at 900 K. The long-range order and cleanliness were confirmed by low energy electron diffraction (LEED). The molecules were deposited via a multi-source thermal evaporator (Unisoku, UE-203C series). The successful deposition of *n*-C_30_D_62_ and *n*-C_32_H_66_ were inspected with the TPD signal of propyl fragments. TPD signals were collected at a heating rate of 0.5 K/s and 2 K/s as described.

**Regents:** *n*-dotriacontane (*n*-C_32_H_66_) (98%) and deuterated triacontane (*n*-C_30_D_62_) (98%) were purchased from Sigma-Aldrich. Octadecylbenzene (OB) was purchased from TCI. All the commercially available reagents were used without further purification unless otherwise specified. 2-dodecylnaphthalene (2-DN) was synthesized as discussed in the following section.

**Synthesis of 2-dodecylnaphthalene**

All the commercially available reagents were purchased from Sigma-Aldrich, TCI or Aladdin (Shanghai) Inc. and used without further purification, unless otherwise specified. Tetrahydrofuran was distilled from sodium/benzophenone under argon.

*Chromatography*

Thin layer chromatography analyses were performed on silica gel Merck 60 F_254_ sheets; detection was conducted by observation under short-wavelength UV (254 nm) and long-wavelength UV (365 nm). Column chromatography was performed using silica gel (Qingdao Haiyang, particle size 0.054-0.074 mm).

*NMR (Nuclear magnetic resonance):*

Nuclear magnetic resonance spectra were recorded on Bruker AVANCE NEO (400 MHz for ^1^H, 100 MHz for ^13^C) spectrometer as solutions in CDCl_3_ at 20 °C.

Chemicals shifts (in ppm) were determined relative to TMS (0.03%(V/V)) as internal reference in deuterated solvents. Spin multiplicities are given with the following abbreviations: s (singlet), brs (broad singlet), d (doublet), dd (doublet of doublet), t (triplet), q (quadruplet), m (multiple) and coupling constants (*J*) quoted in Hz.

*Mass spectra (MS)*

High-resolution mass spectra (HRMS) (positive or negative mode EI: Electron Ionization) were recorded on the Waters GCT Premier Mass Spectrometer with time-of-flight detector.

Synthesis of 2-dodecylnaphthalene

Magnesium shavings (0.22 g, 9.17 mmol), a single chip of iodine, 1 mL dry THF were added into a well-dried 50 mL two-necked flask, the mixture was degassed/argon several times before 10 drops of 1-bromodocecane (1.48 g, 5.94 mmol in 5 mL dry THF) was added. The reaction was initiated by gentle heating and the 1-bromdodecane solution was added dropwise at 50 °C, the mixture was stirred at 50 °C for 3 hours then cooled to room temperature. In another dry 50 mL two-necked flask, 2-bromonaphthalene (0.82 g, 3.96 mmol), Ni(dppp)Cl_2_ (0.02 g, 0.037 mmol), 5 mL dry THF were stirred under an argon atmosphere, the pre-prepared bromododecymagnesium solution was added dropwise while maintaining the temperature below 30 °C, after stirring overnight at room temperature, 2 M aqueous HCl was added dropwise to neutralize the mixture, then 20 mL diethyl ether was added and the organic layer was washed by 40 mL saturated brine, 40 mL*2 distilled water, then the organic layer was collected, dried over anhydrous MgSO_4_, filtrated, concentrated with rotation evaporator, the crude products was purified by column chromatography (SiO_2_, cyclohexane) to obtain the product as white solid. (0.76 g, 66%)

^1^H NMR (400 MHz, CDCl_3_) δ 7.81-7.73 (m, 3H), 7.60 (d, 1H, *J* = 1.7 Hz), 7.45-7.37 (m, 2H), 7.32 (dd, 1H, *J_1_* = 8.4 Hz, *J_2_* = 1.7 Hz, 2.76 ( t，2H, *J* = 7.7 Hz)，1.74-1.64 ( m，2H)，1.42-1.19 (m，18H)，0.88 ( t，3H, *J* = 6.6 Hz). ^13^C NMR (100 MHz, CDCl_3_) δ 140.48, 133.65, 131.92, 127.70, 127.58, 127.46, 127.39, 126.27, 125.78, 124.96, 36.13, 31.93, 31.39, 29.68 (2C), 29.65, 29.61, 29.55, 29.36 (2C), 22.70, 14.12. HRMS (EI-TOF) calculated for [C_22_H_32_] 296.2504, found m/z 296.2497.

**References**

1. Bartels L, Meyer G and Rieder KH et al. Dynamics of electron-induced manipulation of individual CO molecules on Cu(111). *Phys Rev Lett* 1998; **80**: 2004-2007.

2. Kresse G and Furthmüller J. Efficient iterative schemes for ab initio total-energy calculations using a plane-wave basis set. *Phys Rev B* 1996; **54**: 11169-11186.

3. Larsen AH, Mortensen JJ and Blomqvist J et al. The atomic simulation environment-a python library for working with atoms. *J Phys: Condens Matter* 2017; **29**: 273002.

4. Blöchl PE. Projector augumented-wave method. *Phys. Rev. B*. 1994; **50**: 17953-17979 (1994).

5. Perdew JP, Burke K and Ernzerhof M. Generalized gradient approximation made simple. *Phys Rev Lett* 1996; **77**: 3865–3868.

6. Grimme S, Antony J and Ehrlich S et al. A consistent and accurate ab initio parametrization of density functional dispersion correction (DFT-D) for the 94 elements H-Pu. *J Chem Phys* 2010; **132**: 154104.

7. Henkelman G, Uberuaga BP and Jónsson H. A climbing image nudged elastic band method for finding saddle points and minimum energy paths. *J Chem Phys* 2000; **113**: 9901-9904.

8. Henkelman G and Jónsson H. Improved tangent estimate in the nudged elastic band method for finding minimum energy paths and saddle points. *J Chem Phys* 2000; **113**: 9978-9985.

9. Henkelman G and Jónsson H. A dimer method for finding saddle points on high dimensional potential surfaces using only first derivatives. *J Chem Phys* 1999; **111**: 7010-7022.

10. Monkhorst HJ and Pack JD. Special points for Brillouin-zone integrations. *Phys Rev B* 1976; **13**: 5188-5192.

11. Björk J, Stafström S and Hanke F. Zipping up: cooperativity drives the synthesis of graphene nanoribbons. *J Am Chem Soc* 2011; **133**: 14884-14887

12. Yu X, Schwarz P and Nefedov A et al. Structural evolution of water on ZnO(10 -1 0): from isolated monomers via anisotropic H-bonded 2D and 3D structures to isotropic multilayers. *Angew Chem Int Ed* 2019; **58**: 17751-17757.

13. Ren Z, Guo Q and Xu C et al. Surface photocatalysis-TPD spectrometer for photochemical kinetics. *Chin J Chem Phys* 2012; **25**: 507-512.

**Figure S1. Adsorption geometry of *n*-alkanes on Cu(110).** Among the candidate adsorption geometry of *n*-C_10_H_22_ (**a**, lying on the groove; **b**, standing on the groove; **c**, lying on the ridge; **d**, standing on the ridge), the flat-on geometry is the most favorable either on the grooves or ridges. **e,** nc-AFM image of the *n*-dotriacontane molecules and the underpinning copper atoms. (molecules: dz = 130 pm; the substrate: dz = -170 pm; STM offset: -5 mV, 8 pA.) The blue dashed line in **(e)** labels the flat-on *n*-dotriacontane settling on the grooves while the red and yellow dashed lines label the slightly tilt-on molecules whose adsorption sites shift towards the ridges. **f,** Cross-section profile (27 lines averaged) along the bule lines in **(e)**.


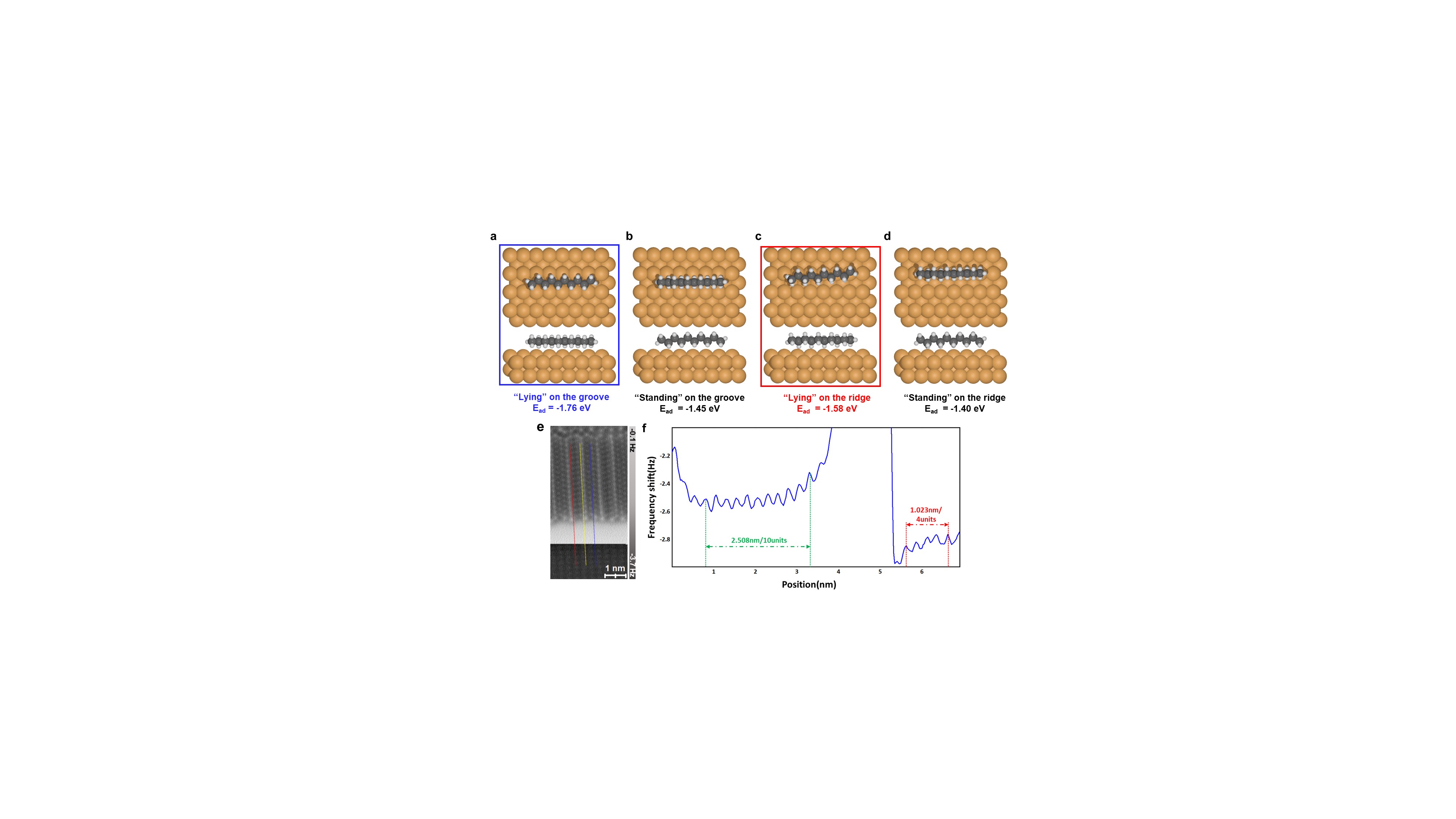


**Figure S2.** **Adsorption geometry of [32]-polyenes on Cu(110). a**, nc-AFM image of the [32]-polyene molecules and the underpinning copper atoms overlapped with DFT simulated results (molecules: dz = 30 pm; the substrate: dz = -170 pm; STM offset: -5 mV, 8 pA.). **b**, Cross-section profile (27 lines averaged) along the polyene molecules for periodicity measurements.


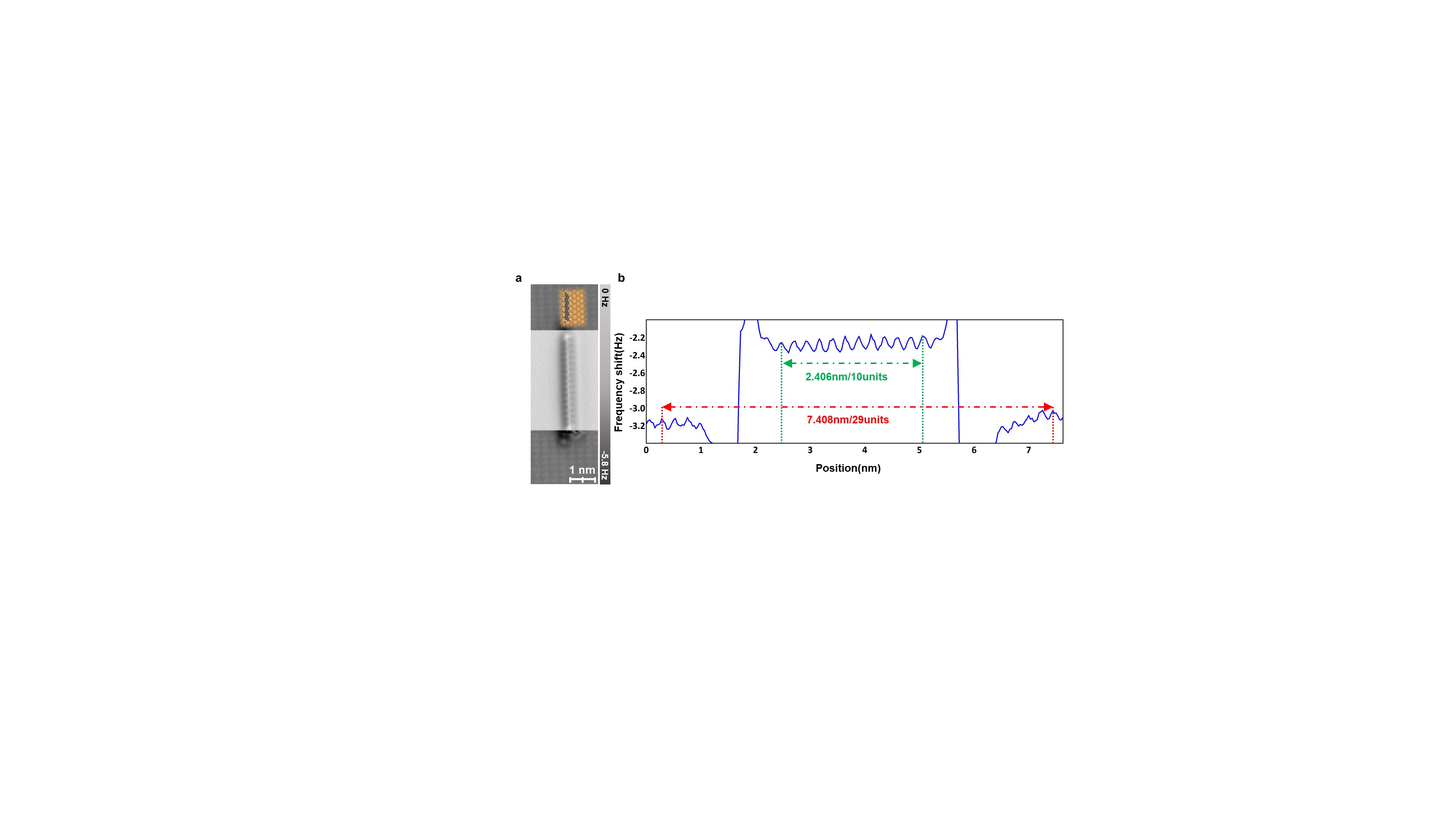


**Figure S3. Transformation in the self-assembly of *n*-C_32_H_66_ molecules. a**, STM image (-1 V, 10 pA) of the self-assembly of intact *n*-C_32_H_66_ molecules. The close-up inset (-5 mV, 100 pA) shows the typical superstructure and the underpinning cooper rows. **b**, STM image (-200 mV, 10 pA) after annealing the sample under 493 K for 15 minutes. The inset (-5 mV, 10 pA) indicates the occurrence of transformed [32]-polyene molecules. **c**, STM image after extra 15 minutes annealing under 493 K where the inset implies the alkenyl homocoupling product. **d**, STM image (-50 mV, 8 pA) after an hour annealing under 493 K.

**
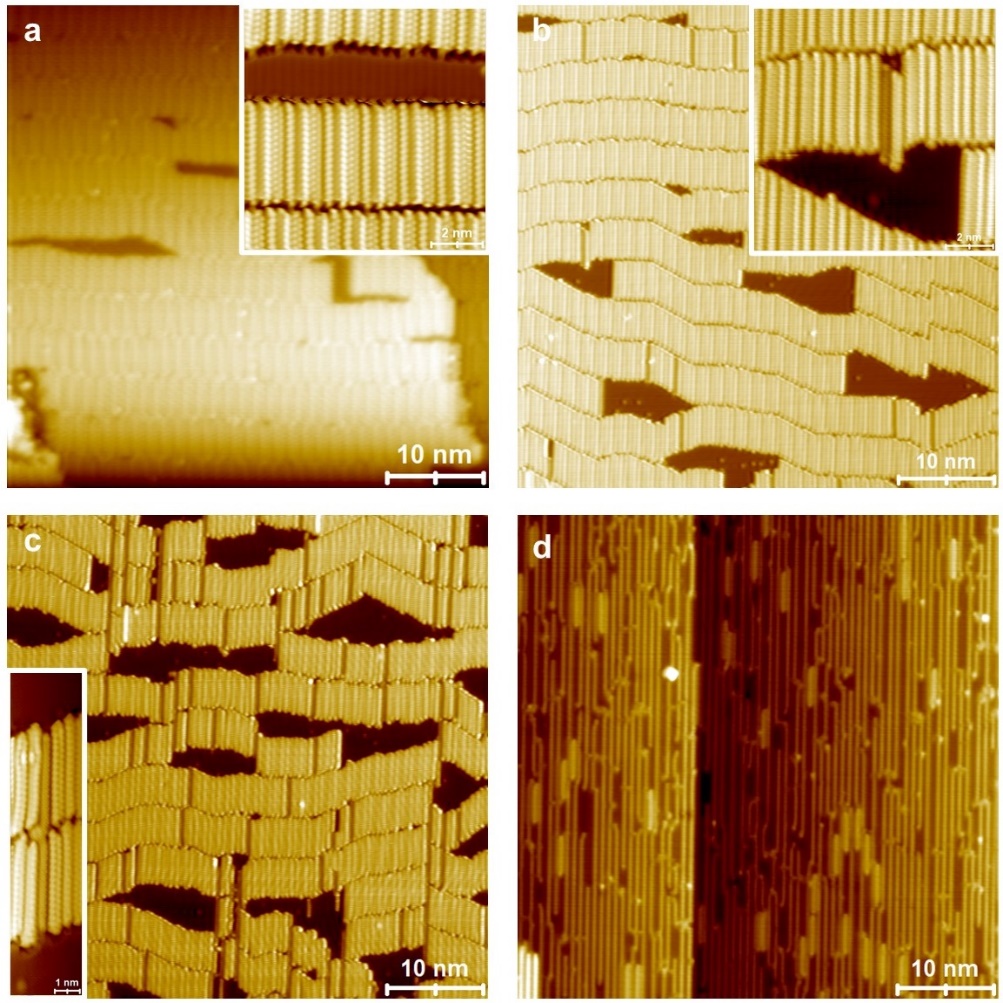
**

**Figure S4.** **Defects in alkenyl homocoupling.** Cis-isomerism of double bonds is the contributing factor of defects observed in the global polymerization of [32]-polyene. The detects appear either between different chains (**a, b,** -50 mV, 25 pA, dz = -80 pm for nc-AFM imaging with respect to the STM offset) or in a single chain (**c, d,** -5 mV, 8 pA, dz = 0 pm for nc-AFM imaging with respect to the STM offset) during the alkenyl homocoupling process. These isomerized knots are also assumed as the cyclization stimuli under higher annealing conditions.

**
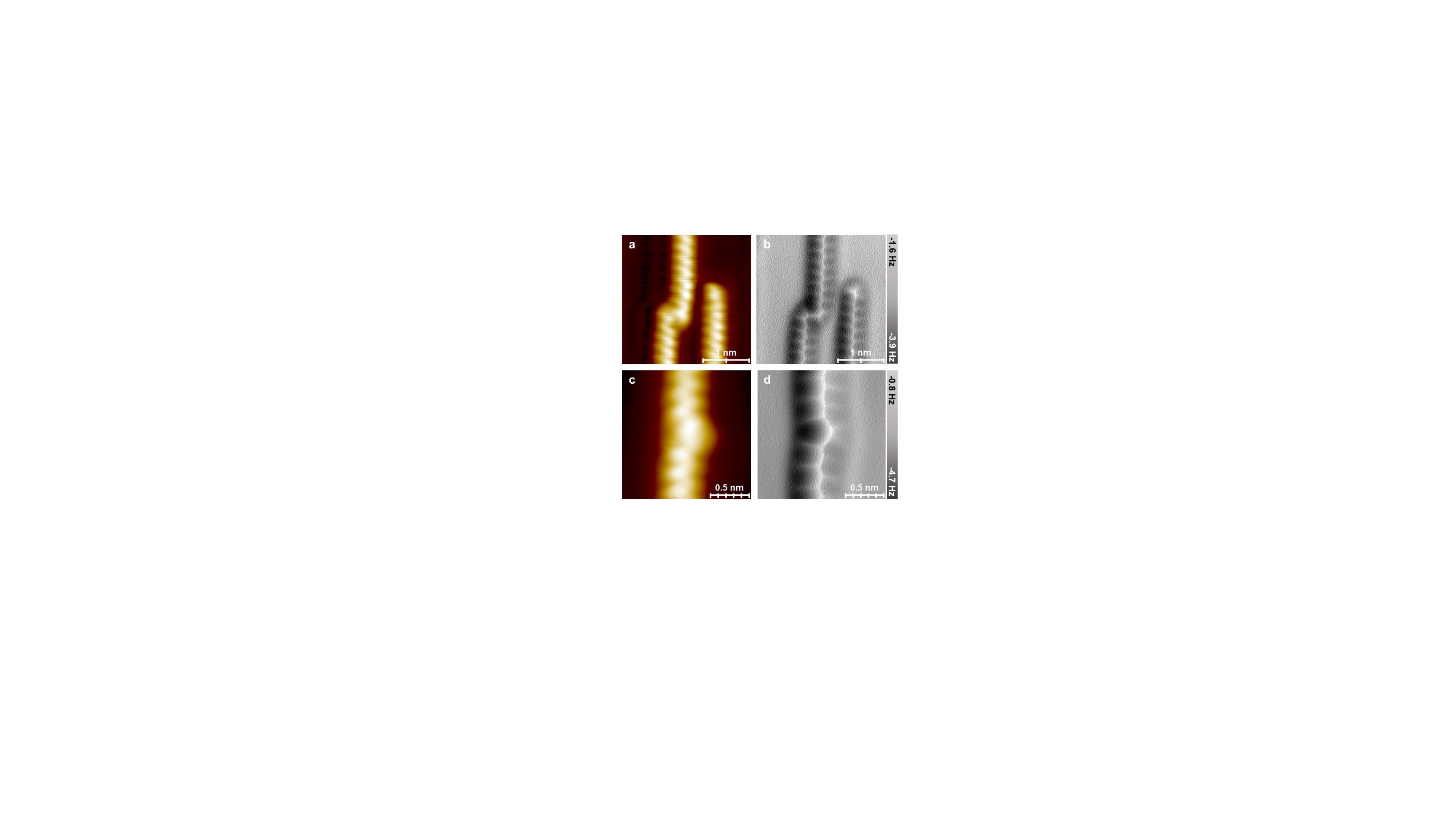
**

**Figure S5. Angle resolved photoemission spectroscopy measurements. a**, STM image of the intact *n*-C_32_H_66_ sample for ARPES measurements (-2 V, 5 pA). **b**, STM image of the polymerized polyene sample for ARPES measurements (-1 V, 100 pA). **c**, STM image of the bare Cu(110) transferred for ARPES measurements (-200 mV, 10 pA). **d**, Fermi surface topology of polymerized polyenes on Cu(110) with a photon energy of 21.2 eV. The ARPES data are symmetrized with respect to *k*_x_ = 0. **e**, Normalized band structure of polymerized polyenes on Cu(110) along Γ-Y direction. The total image was mapped by three cuts. The bands from polyenes are highlighted by white dashed lines, whereas Cu band is marked by orange dashed line. The band dispersion of polymerized polyenes in our experiments seems to be non-linear compared with the reported dispersion of individual polyacetylene chain. The observed non-linearity might be owing to the cross-linking among polyene chains which results in a two-dimensional conducting network.

**
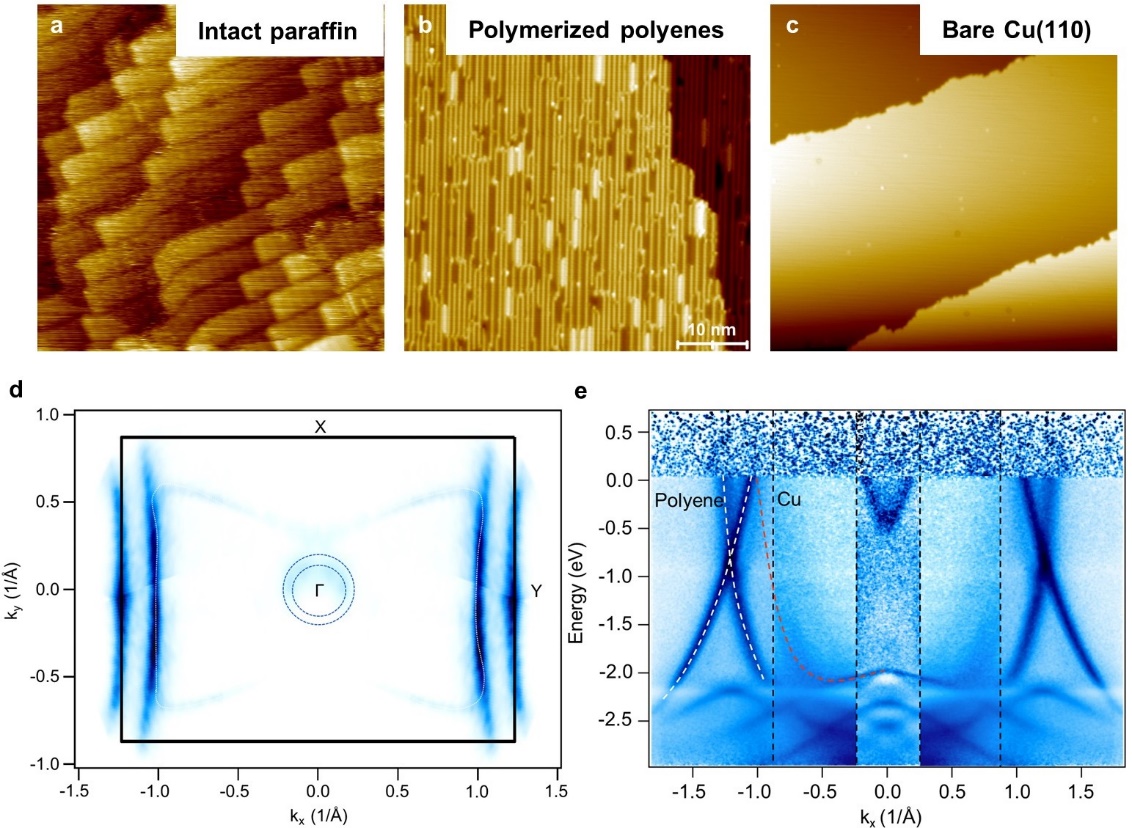
**

**
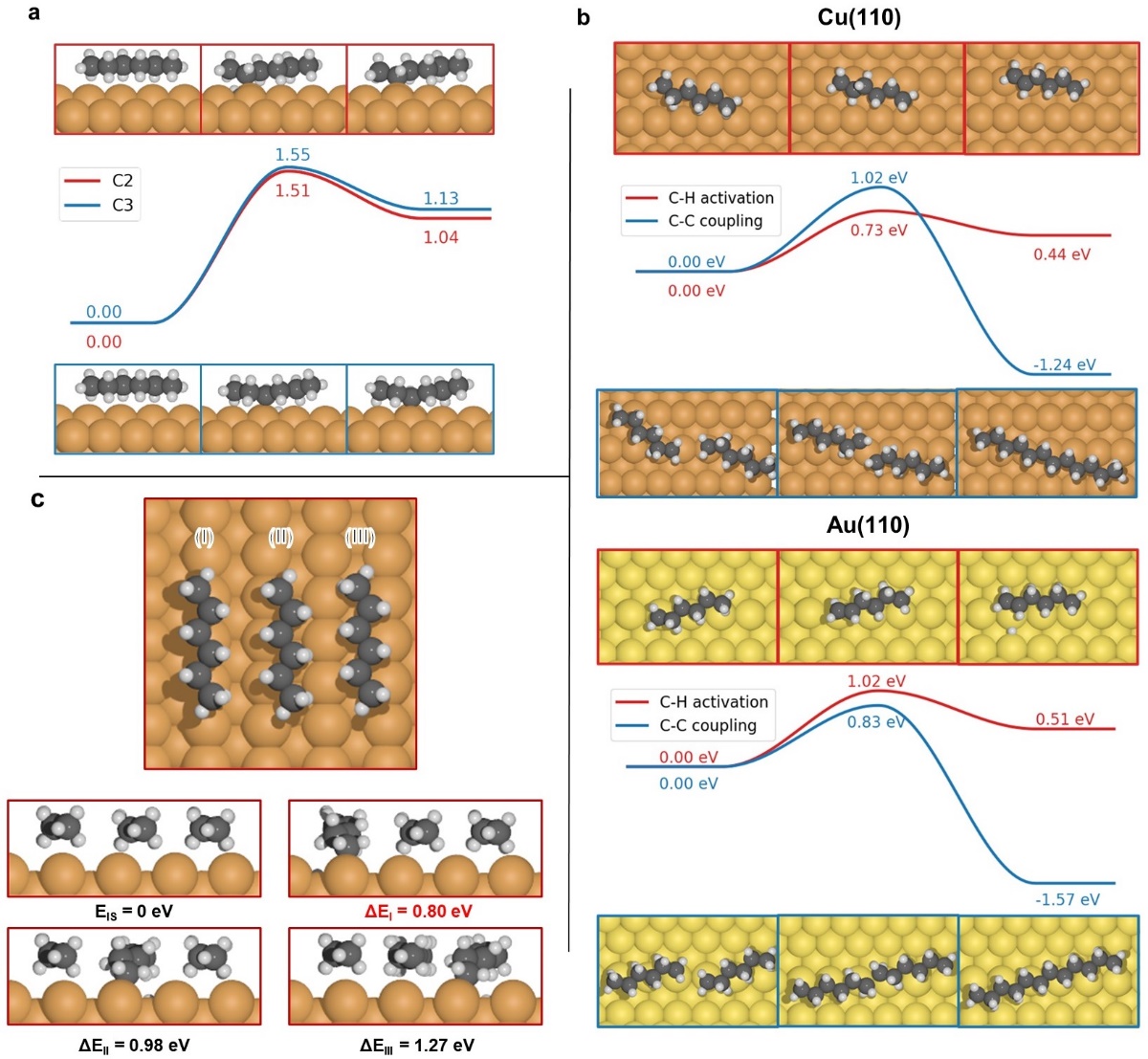
Figure S6. Complementary DFT calculations. a**, Reaction pathways and corresponding energy profiles for the dehydrogenations at C2 (red) and C3 (blue) site. **b**, The comparison between the 2nd dehydrogenation step (S2 to S3) and the homocoupling of 1-hexyl radicals on both Cu(110) and Au(110). The Cu, C and H atoms are represented by the yellow, grey and white circles, respectively. Units for all profiles are in eV. The C−H activation at the C2 site of the 1-hexyl (**E_TS2_**) on Cu(110) is 0.73 eV, which is lower than that of the C−C coupling (1.02 eV) between two 1-hexyl radical groups on Cu(110). Meanwhile, the C−H activation at the C2 site of the 1-hexyl on Au(110) is 1.02 eV, which is higher than that of the C−C coupling (0.83 eV) pathway. **c**, Self-assembled structure of the linear alkanes and the reaction pathway for the 1st dehydrogenation of the alkane adsorbs on the ridge of Cu(110) (I). The results suggest that the dehydrogenation at (I) are more energetically favorable. The difference in the thermodynamics of terminal C-H activations is ascribed to the distance between the C atoms and the Cu catalytic sites. The *n*-hexane molecule adsorbed at (I) exhibits the shortest C−Cu distance of 3.26 Å, while the C−Cu distance for the molecule adsorbed at (III) is 4.07 Å.

**
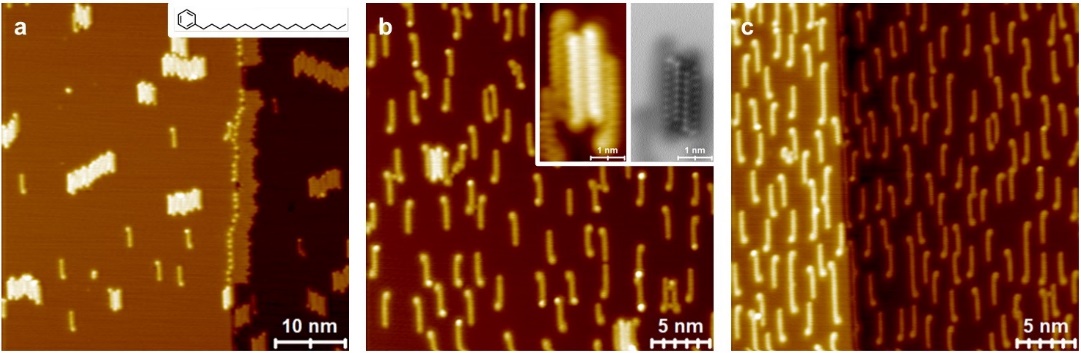
Figure S7.** **On-surface transformation of Octadecylbenzene. a**, Chemical formula and STM image (-50 mV, 8 pA) after room temperature molecular deposition. Although there are already some isolated molecules distributed on the surface, extra room temperature annealing would not trigger overall transformation of OB molecules. **b**, STM image (-50 mV, 8 pA) after annealing under 393 K for 30 minutes. The inset presents the nc-AFM image and the according STM image (-5 mV, 8 pA) of the mixture with both intact molecules and transformed molecules at an imaging height of dz = 130 pm. The extra phenyl group had little impact on the adsorption of transformed polyenes. Interestingly, some of the molecules exhibited a bright ending in the STM image which might be the case of an unreacted methylene terminal. **c**, STM image (-50 mV, 8 pA) after annealing at 453 K for 30 minutes where alkenyl homocoupling products prevail.

**Figure S8. On-surface transformation of 2-dodecylnaphthalene. a**, Chemical formula and STM image (-5 mV, 8 pA) after 2 minutes room temperature molecular deposition of 2-dodecylnaphthalene. The naphthyl substituents within the self-assembly aligned regularly in a saw-like shape and modified the packing direction. The insets present the Laplacian-filtered nc-AFM image and the according STM image (-5 mV, 8 pA) of intact 2-DN molecules at an imaging height of dz = 130 pm with respect to the STM offset. The naphthyl substituents were ambiguously visualized at the imaging height of alkyl chains which implied a decreased adsorption height. **b**, STM images (-200 mV, 8 pA) after 8 hours room temperature annealing where the global transformation of 2-DN is observed.


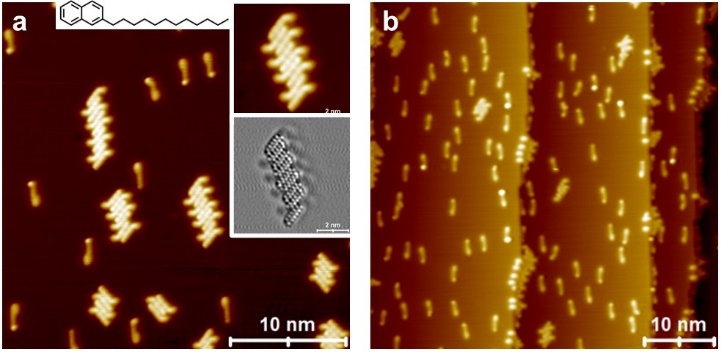


**Figure S9. On-surface transformation of 4,4''-didodecyl-5'-(4-dodecylphenyl)-1,1':3',1''-terphenyl (DDT) on Cu(110). a,** Chemical formula of DDT. **b, c,** STM and the corresponding nc-AFM images of a partially transformed DDT molecule after depositing DDT molecules onto the Cu(110) substrate held at 350K. **d, e,** STM and the corresponding nc-AFM images of a partially transformed molecule where the olefination is found to initiate from the phenyl ring site as marked by the dashed yellow line. The phenyl-ring-like structure marked by dashed red line is occasionally observed on the surface as shown in **f** and **g** which is the by-products synthesized.


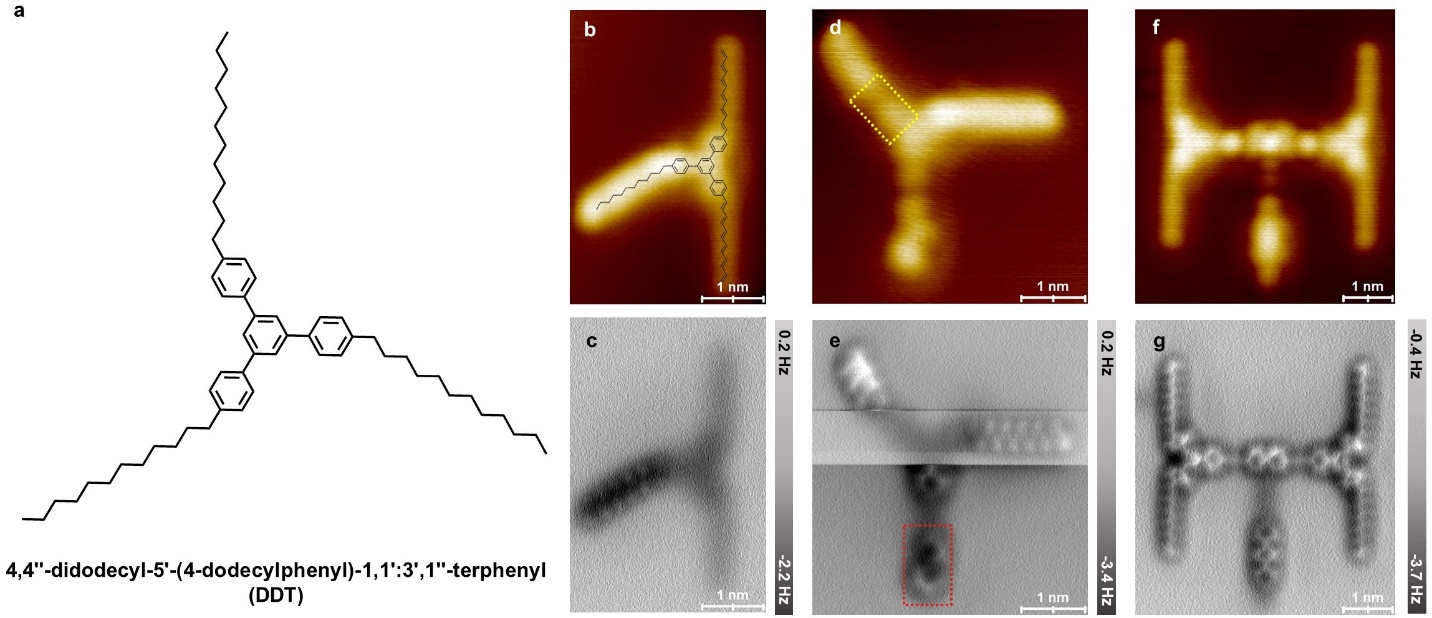


**Figure S10. On-surface transformation of DDT on Cu(111). a,** Chemical formula of DDT. b, c, STM and the corresponding nc-AFM images of the self-assembly of DDT molecules after room temperature deposition onto Cu(111) surface. d, e, STM and the corresponding nc-AFM images of a transformed molecule after annealing the sample under 390K for half an hour.


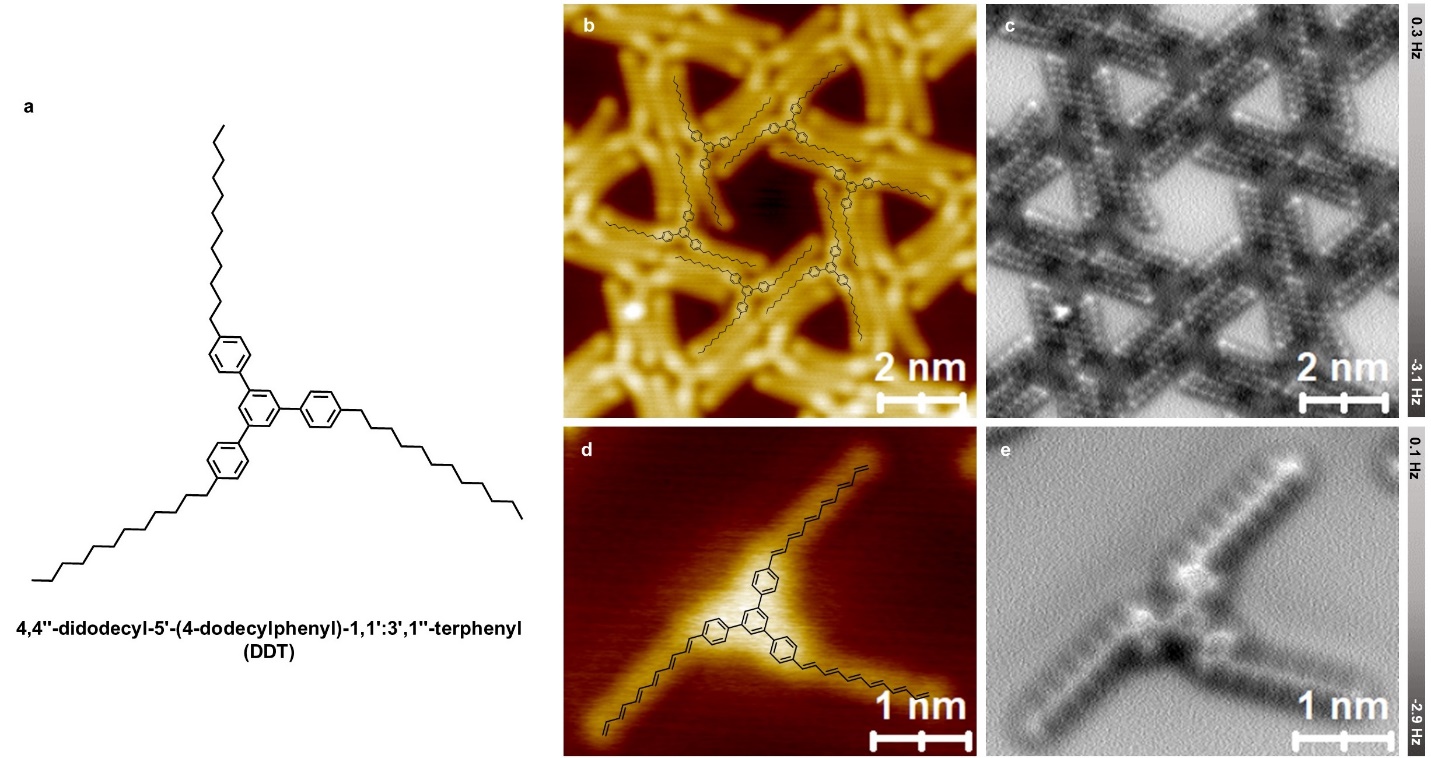

Supplement: nwab093_Supplemental_File [file nwab093_supplemental_file.docx]
